# Activity of Cardiomyocyte Type 3 Deiodinase After Myocardial Infarction Influences Cardiac Recovery in Females

**DOI:** 10.1210/endocr/bqaf181

**Published:** 2025-12-12

**Authors:** Maigen Bethea, Tyler Cook, Preston Stafford, Leslie Knaub, Maria Elena Martinez, Bjoern Schniedewind, Uwe Christians, Jasmine Jay Hendrix, Luisa Mestroni, Sharon Graw, Anis Karimpour-Fard, Matthew R G Taylor, Ronald J Vagnozzi, Arturo Hernandez, Rebecca Scalzo, Darleen A. Sandoval, Silvania da Silva Teixeira

**Affiliations:** Department of Pediatrics, Section of Nutrition, University of Colorado Anschutz Medical Campus, Aurora, CO 80045, USA; Department of Pediatrics, Section of Nutrition, University of Colorado Anschutz Medical Campus, Aurora, CO 80045, USA; Division of Cardiology, Department of Medicine, University of Colorado School of Medicine (UCSOM), Aurora, CO 80045, USA; Division of Endocrinology, Department of Medicine, University of Colorado School of Medicine (UCSOM), Aurora, CO 80045, USA; Department of Medicine, Rocky Mountain Regional Veterans Affairs Medical Center, Aurora, CO 80045, USA; Center for Molecular Medicine, MaineHealth Institute for Research, MaineHealth, Scarborough, ME 04074, USA; Department of Anesthesiology, University of Colorado School of Medicine, Aurora, CO 80045, USA; iC42 Clinical Research and Development Clinical Mass Spectrometry Service Center, University of Colorado School of Medicine, Aurora, CO 80045, USA; Department of Anesthesiology, University of Colorado School of Medicine, Aurora, CO 80045, USA; iC42 Clinical Research and Development Clinical Mass Spectrometry Service Center, University of Colorado School of Medicine, Aurora, CO 80045, USA; Department of Pediatrics, Section of Nutrition, University of Colorado Anschutz Medical Campus, Aurora, CO 80045, USA; Division of Cardiology, Department of Medicine, University of Colorado School of Medicine (UCSOM), Aurora, CO 80045, USA; Division of Cardiology, Department of Medicine, University of Colorado School of Medicine (UCSOM), Aurora, CO 80045, USA; Division of Cardiology, Department of Medicine, University of Colorado School of Medicine (UCSOM), Aurora, CO 80045, USA; Division of Cardiology, Department of Medicine, University of Colorado School of Medicine (UCSOM), Aurora, CO 80045, USA; Division of Cardiology, Department of Medicine, University of Colorado School of Medicine (UCSOM), Aurora, CO 80045, USA; Center for Molecular Medicine, MaineHealth Institute for Research, MaineHealth, Scarborough, ME 04074, USA; Graduate School for Biomedical Science and Engineering, University of Maine, Orono, ME 04469, USA; Department of Medicine, Tufts University School of Medicine, Boston, MA 02111, USA; Division of Endocrinology, Department of Medicine, University of Colorado School of Medicine (UCSOM), Aurora, CO 80045, USA; Department of Medicine, Rocky Mountain Regional Veterans Affairs Medical Center, Aurora, CO 80045, USA; Department of Pediatrics, Section of Nutrition, University of Colorado Anschutz Medical Campus, Aurora, CO 80045, USA; Department of Pediatrics, Section of Nutrition, University of Colorado Anschutz Medical Campus, Aurora, CO 80045, USA

**Keywords:** type 3 deiodinase, myocardial infarction, sex differences, thyroid hormone, cardiac recovery

## Abstract

Thyroid hormone (TH) is essential for cardiovascular function, and women are disproportionately affected by TH disorders and experience worse outcomes following myocardial infarction (MI). However, the role of sex-specific TH regulation in post-MI cardiac recovery remains poorly understood. We investigated TH homeostasis and type 3 deiodinase (D3) activity, an enzyme that inactivates TH, in male and female C57BL/6 mice following MI. Using cardiomyocyte-specific D3-deficient (*Dio3*^ΔHeart^) mice, we investigated how impaired TH inactivation influences cardiac function and mitochondrial respiration. We also examined *DIO3* messenger RNA expression, which encodes the D3 enzyme, in left ventricular (LV) tissue from human donors with nonfailing (NF) hearts or ischemic cardiomyopathy (ICM). Four weeks post MI, wild-type female mice exhibited sustained cardiac D3 activity, which effectively limited 3,5,3′-triiodothyronine (T3) levels in the LV. In contrast, *Dio3*^ΔHeart^ females, lacking cardiomyocyte D3, showed impaired systolic recovery, elevated LV thyroxine and T3 levels, and reduced fatty acid–supported mitochondrial respiration, effects not observed in *Dio3*^ΔHeart^ males. Similarly, *DIO3* expression was selectively upregulated in LV tissue from women with ICM, but not in men. These findings identify *DIO3* as a key protective mechanism in females that limits T3-induced metabolic stress and preserves mitochondrial function after MI, revealing a sex-dependent pathway with therapeutic relevance for cardiac recovery.

Although women have a lower overall prevalence of cardiovascular disease than men, they face a disproportionately higher risk of heart failure (HF) and a 20% increased mortality following myocardial infarction (MI) (1). Multiple sex-specific risk factors, such as smoking, metabolic syndrome, and the hormonal changes of menopause, have been linked to these disparities (2). However, a critical and underexplored contributor may lie in differences in thyroid hormone (TH) regulation. Women are significantly more likely to develop thyroid disorders than men, and TH is essential for cardiovascular function, influencing heart rate, contractility, vascular tone, and mitochondrial metabolism (3, 4). Despite this, the role of sex-specific TH regulation following MI remains poorly understood.

TH activity is tightly regulated not only by systemic production from the thyroid gland but also through local activation and inactivation by a family of deiodinase enzymes. Type 1 (D1) and type 2 (D2) deiodinases activate TH by converting thyroxine (T4) into its biologically active form, 3,5,3′-triiodothyronine (T3). In contrast, type 3 deiodinase (D3) inactivates T4 and T3, converting them to reverse T3 (3,3,5′-triiodothyronine; rT3) and 3,3′-diiodothyronine (T2), respectively, thereby limiting TH signaling at the tissue level (5).

Following cardiac injury, D3 is significantly upregulated in the left ventricle (LV), and its activity correlates with disease severity (5-8). This suggests D3 plays an important role in the local regulation of TH during the cardiac stress response (6). However, whether cardiac D3 upregulation post MI represents a protective adaptation or contributes to pathological remodeling and HF progression remains unclear (7). Furthermore, it is unknown whether D3 plays a role in the sex-specific differences in cardiac outcomes observed post MI. To date, no studies have systematically examined how TH metabolism differs between male and female hearts following MI, nor whether D3-mediated TH inactivation promotes or impairs recovery in a sex-dependent manner.

In this study, we investigated the role of D3 in post-MI cardiac recovery in male and female mice and extended our studies to humans by analyzing *DIO3* expression, which encodes the D3 enzyme, in LV tissue from individuals with ischemic cardiomyopathy (ICM) and from nonfailing (NF) donor hearts. We found that female mice exhibit a sustained, 2-fold increase in LV D3 activity post MI, while males show only a transient rise. This D3 upregulation in females occurred independently of changes in circulating T3 levels but was accompanied by reduced tissue T3 in the LV. Using a cardiomyocyte-specific D3-deficient mouse model, we show that disruption of D3 activity impairs functional recovery and lipid-supported mitochondrial respiration in female, but not male, hearts. Consistent with these preclinical findings, *DIO3* expression was selectively upregulated in the LV of women with ICM, while *DIO3* levels remained unchanged in men with ICM compared to NF controls. These findings identify D3 as a key sex-specific regulator of cardiac TH availability and metabolic adaptation after MI and offer new insights into mechanisms contributing to worse post-MI outcomes in women.

## Materials and Methods

### Sex as a Biological Variable

This study included both male and female C57BL/6 mice to investigate sex-specific differences in TH regulation and cardiac recovery following MI. A cardiomyocyte-specific D3 deficiency model (*Dio3*^ΔHeart^) was used to assess the effect of impaired TH inactivation. While both sexes were initially evaluated, experiments assessing infarct scar remodeling, cardiac hypertrophy, and mortality were performed exclusively in *Dio3*^ΔHeart^ females; therefore, conclusions regarding sex specificity in structural remodeling are limited to females. This decision was based on preliminary data indicating that loss of cardiomyocyte D3 activity primarily impairs cardiac and metabolic function in female mice. Human LV tissue samples both from male and female donors were analyzed to evaluate sex-specific *DIO3* expression in ICM.

### Animals and Housing

The study used 8-week-old male and female C57BL/6 mice, including a cardiomyocyte-specific D3 deficiency model (*Dio3*^ΔHeart^) maintained on a C57BL/6 genetic background. Mice were housed in a temperature-controlled (22 °C), light-controlled (14/10-hour light/dark cycle) vivarium with ad libitum access to water and standard rodent chow.

### Generation of a Type 3 Deiodinase–Deficient Mouse

*Dio3* encodes the enzyme type 3 deiodinase (D3), which inactivates THs. To generate *Dio3*^ΔHeart^ mice, we crossed αMHC-MerCreMer transgenic mice (Jackson Laboratory No. 005650, RRID: IMSR_JAX:005650) (8) with *Dio3*^SECIS f/f^ mice (9). The αMHC-MerCreMer transgene directs the expression of tamoxifen-inducible Cre recombinase (MerCreMer), specifically in adult cardiac myocytes under the control of the cardiac-specific α-myosin heavy chain promoter (*Myh6*). The *Dio3*^SECIS f/f^ mice carry a floxed selenocysteine insertion sequence (SECIS) within the *Dio3* gene (9). The SECIS sequence is crucial for incorporating selenocysteine into the catalytic center of the D3 enzyme; thus, its excision results in a truncated and inactive D3 protein. Mice used in this study were wild-type (WT) or homozygous for floxed *Dio3* and heterozygous for α-MHC-MerCreMer (^α*Myh6*Mer-Cre-Mer+/−^). Given that α*Myh6*^Cre+/−^ can induce transient cardiac and off-target effects following tamoxifen activation (10), we used heterozygous MerCreMer mice as controls. Specifically, homozygous floxed *Dio3*^ΔHeart^ (*Dio3*^SECIS f/f^/α*Myh6*^Mer-Cre-Mer+/−^) mice were compared to their WT heterozygous MerCreMer^+/−^ littermate controls (WT/α*Myh6*^Mer-Cre-Mer+/−^) in all experiments. This approach ensured that the observed effects were due to the deletion of the SECIS, leading to a truncated, nonfunctional D3 protein, rather than any unintended Cre-mediated off-target effects. Additionally, to minimize the effects of Cre-loxP recombination and tamoxifen-induced toxicity, a 4-week recovery period was implemented after tamoxifen administration. At age 8 weeks, both *Dio3*^ΔHeart^ and control mice received tamoxifen (1.5 mg/day) for 3 consecutive days to induce Cre-mediated recombination. Successful targeting of the SECIS sequence in *Dio3*^SECIS f/f^ mice was confirmed by evaluating D3 enzymatic activity in cardiac tissue. It is important to note that complete ablation of D3 activity was not expected, given that the heart consists of multiple cell types (11), including fibroblasts and endothelial cells, which express *Dio3* and may be regulated following MI (12-14).

### Myocardial Infarction

MI was induced in 12-week-old control and *Dio3*^ΔHeart^ mice of both sexes, as previously described (15, 16). Briefly, mice were anesthetized with isoflurane (3% induction, 1.5% maintenance; medical air) and ventilated (120 breaths/min, 0.40 cc tidal volume) via oral intubation with a 23-gauge intravenous cannula. Bupivacaine (1-2 mg/kg, subcutaneously) was administered at induction, and buprenorphine SR (1.0 mg/kg, subcutaneously) was given preoperatively and repeated 72 hours post surgery for analgesia. A left thoracotomy was performed through the fourth intercostal space, and the pericardium was opened to expose the heart. The left anterior descending artery was isolated and permanently ligated approximately 2 mm distal to its origin using an 8-0 silk suture. Successful infarction was confirmed by a color change in the myocardium from pink to pale. The chest cavity was sutured, and mice were euthanized at 2, 4, or 12 weeks post MI for further analysis. Sham and MI surgeries within the same cohort were randomized to minimize bias.

### Echocardiography

Cardiac function was assessed by transthoracic echocardiography at baseline and subsequently at 2, 4, and 12 weeks post MI, as previously described (16). Briefly, mice were anesthetized with 3% isoflurane and maintained with 1.5% isoflurane throughout the procedure. Echocardiographic parameters were grouped into 3 categories: (i) cardiac function (ejection fraction [EF], and fractional shortening [FS]); (ii) LV geometry and remodeling (LV internal diameter [LVID], and LV volume [LVVol]); and (iii) LV wall thickness (LV anterior wall thickness [LVAW], and LV posterior wall thickness [LVPW]). LVID and LVVol were analyzed as the percentage change from each animal's individual baseline to account for sex-related differences in body size. Percentage change was calculated as ((post-MI value − baseline value)/baseline value) × 100. This normalization approach was applied both for 2- and 4-week post-MI assessments. All images were analyzed by a blinded observer to minimize bias. To ensure the accurate evaluation of post-MI cardiac function, outliers in the MI group with EF values significantly deviating from the group mean and displaying an EF exceeding their baseline measurement were excluded from analysis.

### Histological Assessment of Scar Size

Post-MI females were anesthetized using isoflurane inhalation (to effect) and euthanized via cervical dislocation. Hearts were perfused with 200 to 300 μL of 1-M KCl solution via apical cardiac puncture using a 26-gauge insulin needle. Hearts were excised and rinsed in ice-cold 1× HBSS, then transferred to 4% paraformaldehyde in phosphate-buffered saline (PBS) and incubated overnight at 4 °C with slight agitation. Tissues were washed 3× in cold PBS, then transferred to 30% sucrose in PBS and incubated overnight at 4 °C with slight agitation for cryoprotection. Following cryoprotection, hearts were embedded in Optimal Cutting Temperature (OCT; Tissue-Tek) compound and flash-frozen in 2-methlybutane chilled on liquid nitrogen. Frozen tissue in OCT was sliced and mounted on slides at a thickness of 7 μM using a Leica CM1860 cryostat. Slides were immersed in Picrosirius red staining solution for 1 hour, then rinsed 3 times in 5% acetic acid in H_2_O for 5 minutes. The slides were finally rinsed 3 times in 100% ethanol for 5 minutes and coverslip mounted in organo/limonene mounting media. Stitched brightfield images of whole heart sections were collected at 20× magnification using an Olympus VS200 Slide Scanning Microscope. Images were analyzed by a blinded observer using QuPath to determine scar size as a percentage of LV perimeter. Animals with no visible MI were excluded from analysis.

### Type 3 Deiodinase Enzymatic Activity

D3 enzymatic activity was assessed as described previously (17). Briefly, tissues were homogenized in a buffer (10 mM Tris-HCl, 0.25 M sucrose, pH 7.4), with volumes adjusted by protein content to maintain deiodination below 40%. Homogenates were incubated with 2 nM of 125I-labeled T3 at 37 °C for 1 hour in the presence of 1-mM propylthioracyl, and deiodination was quantified by measuring the amount of 125I-T2 produced after separation by paper chromatography (18).

### Thyroid Hormone Determination

TH plasma and tissue concentrations of T4, T3, and rT3 were measured using a validated high-performance liquid chromatography–tandem mass spectrometry (LC-MS/MS) assay established at the University of Colorado Clinical Mass Spectrometry Service Center (iC42 Clinical Research and Development). All reference compounds, as well as their corresponding isotope-labeled internal standards, were from Cayman Chemicals. Data analysis was performed with Sciex Analyst software (v1.7.3, Sciex). The analytical measuring range for T4, T3, and rT3 was 0.025 to 100 ng/mL. Intrabatch and interbatch trueness were within ±15% and imprecision less than 15% (coefficient of variance) for each batch. There was no statistically significant carry-over, matrix interferences, or relative matrix effect.

### RNA Isolation, Complementary DNA Synthesis, and Quantitative Polymerase Chain Reaction

Tissues were homogenized in TRIzol reagent, followed by phase separation via centrifugation. The aqueous phase was then processed using the RNeasy Kit (Qiagen, 74104) for total RNA extraction and purification, following the manufacturer's instructions. Complementary DNA (cDNA) synthesis was performed using the iScript cDNA Synthesis Kit (Bio-Rad, 1708890). Gene expression analysis was conducted using quantitative polymerase chain reaction with the TaqMan Multiplex Master Mix (Thermo Fisher Scientific, 4461882). TaqMan gene-specific probes were purchased from Thermo Fisher Scientific, and relative messenger RNA (mRNA) levels were quantified using the ΔCt method, with Rpl4 (Mm05781370_g1) as the endogenous control. The following gene-specific TaqMan assays were used: *Dio3* (Mm00548953_s1), *Dio2* (Mm00515664_m1), *Mct8* (Mm00486204_m1), and *Mct10* (Mm00661045_m1).

### Mitochondrial Respiration

Mitochondrial respiration was measured using the Oroboros Oxygraph-2k, following protocols established for cardiac fibers (19-21), with laboratory-specific modifications (MiR06 buffer; inclusion of blebbistatin; cytochrome *c* acceptance <5%). After heart removal, the LV fibers were separated, permeabilized with saponin, and washed in a respiration buffer (MiR06). Fibers were collected from the adjacent noninfarcted region bordering the infarct (peri-infarct zone) of the LV to capture viable but metabolically stressed myocardium (21). Approximately 0.8 to 1 mg of wet tissue was used per chamber, and oxygen flux was expressed as pmol O₂·s⁻¹·mg⁻¹ wet tissue. Fibers (0.8-1 mg) were placed in MiR06 containing blebbistatin, and oxygen levels were maintained between 250 and 400 µM. Respiration was assessed using carbohydrate-linked substrates (pyruvate, malate, glutamate, succinate) and lipid-linked substrates (palmitoylcarnitine, malate, glutamate, succinate). Maximal uncoupling was achieved with FCCP to determine electron transport system capacity, and cytochrome c was added to verify mitochondrial outer membrane integrity (samples were accepted if respiration increased by <5%). Respiration ratios for both substrate types were calculated for LEAK/OXPHOS and OXPHOS/Uncoupled states.

### Human Cardiac Tissue RNA Sequencing and Data Processing

RNA was extracted from 327 human heart samples obtained through the University of Colorado, Division of Cardiology, cardiac tissue bank, a long-standing, institutional review board–approved protocol for collecting cardiac tissue at the time of cardiac transplantation or LV assist device implantation. The dataset included 187 samples from individuals with ICM (29 females, 158 males) and 140 from NF donor hearts (79 females, 61 males). Bulk LV tissue (∼30 mg) was homogenized, and RNA was extracted using RNeasy Mini Kits (Qiagen). RNA sequencing (RNA-seq) of poly-A–enriched RNA was performed by the Broad Institute of MIT and sequenced on the Illumina HiSeq 4000 platform (Illumina) to a target depth of 40 million or more 2 × 101 bp paired-end reads. Raw sequencing data were processed using the TOPMed MESA RNA-seq pipeline, which includes alignment, quality control, and transcript quantification (https://topmed.nhlbi.nih.gov/sites/default/files/TOPMed_RNAseq_pipeline_flowchart_COREyr2.pdf). Reads were aligned to the GRCh38 reference genome using STAR, quantified at the gene level using RNA-SeQC v2, and annotated using the GENCODE v34 reference. Pipeline details are available at: https://github.com/broadinstitute/gtex-pipeline/blob/master/TOPMed_RNAseq_pipeline.md.

Regarding the distribution of tested human tissue samples by sex, the predominance of male samples reflects the population undergoing cardiac transplantation. Males are transplanted at a significantly higher rate than females, both at our center and nationwide. According to the United Network for Organ Sharing (UNOS), males account for 71.8% of all heart transplants in 2024 and 72.0% across all years. Thus, our dataset composition mirrors national transplant demographics. All analyzed samples correspond to bulk LV tissue.

### Statistical Analysis

All statistical analyses were performed using GraphPad Prism version 10 (GraphPad Software; RRID: SCR_002798). For murine studies, 3-way analysis of variance (ANOVA) was used to assess interactions among sex, genotype, and MI. When statistically significant interactions were detected, multiple comparisons were performed using the 2-stage linear step-up procedure of Benjamini, Krieger, and Yekutieli to control the false discovery rate. Two-way ANOVA was used when 2 factors were analyzed, and the Tukey post hoc test was applied where appropriate. Unpaired 2-tailed *t* tests were used for direct pairwise comparisons. Survival analyses were conducted using the log-rank (Mantel-Cox) test. Data are presented as mean ± SEM, and statistical significance was defined as *P* less than .05.

For human transcriptomic analyses, *DIO3* expression (log₂ TPM) was compared between NF and ICM hearts within each sex using the Wilcoxon rank sum test. Additionally, the association between age and *DIO3* expression was assessed within each group using Spearman correlation analysis. Separate analyses were performed for female and male cohorts.

### Study Approval

Animal studies were conducted in accordance with the National Institutes of Health (NIH) guidelines and were approved by the University of Colorado Anschutz Medical Campus Institutional Animal Care and Use Committee (IACUC protocol No. 1135).

## Results

### Myocardial Infarction Induces Sex-Specific Alterations in Cardiac Thyroid Hormone Regulation

We first determined sex differences in cardiac function before and after MI in WT mice (Table 1). Following MI, male and female mice both exhibited a substantial decline in systolic function, as indicated by significantly reduced EF and FS at all post-MI time points relative to baseline. These reductions were comparable between sexes, indicating similar systolic impairment in our C57BL/6 model, though outcomes may vary depending on genetic background and experimental design (22-24). Consistent with previous studies (25, 26), we observed clear sex-specific differences in LV structural remodeling. Male mice showed consistently greater LV dilation after MI, as indicated by larger percentage changes from individual baseline values in LV end-diastolic and end-systolic volumes (LV Vol;d and LVVol;s) and internal diameters (LVID;d and LVID;s) compared to females at all time points. These differences were evident by 4 weeks post MI and persisted through 12 weeks. Despite these structural differences, heart rate (HR) remained stable across groups, and no consistent sex-based differences in cardiac output (CO) were noted. Collectively, these findings suggest that while MI induced similar systolic dysfunction in both sexes in our C57BL/6 model, male mice experienced more pronounced adverse LV remodeling, highlighting sex-specific differences in the cardiac response to injury (see Table 1). To determine whether sex-dependent differences in ventricular remodeling were associated with changes in cardiac mass, we measured body weight (BW), heart weight (HW), and HW/BW ratios in WT male and female mice 12 weeks after MI (Table 2). MI significantly increased HW and HW/BW ratios in both sexes compared with sham, consistent with post-infarction hypertrophy. Although females had lower absolute BWs and HWs than males, the relative HW/BW response to MI was similar between the sexes, indicating a comparable degree of cardiac hypertrophy.

**Table 1.** Echocardiographic assessment of left ventricular structure and function in male and female mice at baseline and at 4, 8, and 12 weeks after myocardial infarction

	Male (n = 7)				Female (n = 5)			
Parameters	Baseline	4 wk MI	8 wk MI	12 wk MI	Baseline	4 wk MI	8 wk MI	12 wk MI
EF, %	54.07 ± 0.68	22.18 ± 4.42*^a^*	22.24 ± 3.83*^a^*	20.30 ± 3.15*^a^*	52.96 ± 2.11	22.07 ± 2.61*^a^*	22.57 ± 2.51*^a^*	18.96 ± 1.63*^a^*
FS, %	27.51 ± 0.43	10.40 ± 2.13*^a^*	10.39 ± 1.85*^a^*	9.40 ± 1.49*^a^*	26.64 ± 1.32	10.12 ± 1.22*^a^*	10.45 ± 1.23*^a^*	8.64 ± 0.77*^a^*
LV Vol;d, μL	66.34 ± 2.43	210.059 ± 61.43*^a^*	217.546 ± 43.10*^a^*	216.290 ± 60.33*^a^*	53.12 ± 2.05*^b^*	118.465 ± 23.70*^a,b^*	151.394 ± 18.53*^a,b^*	138.101 ± 19.87*^a,b^*
LV Vol;s, μL	30.47 ± 1.21	463.685 ± 138.83*^a^*	467.303 ± 97.08*^a^*	476.633 ± 123.80*^a^*	25.13 ± 1.93*^b^*	263.124 ± 42.13*^a,b^*	320.447 ± 45.71*^a,b^*	319.023 ± 52.47*^a,b^*
LVID;d, mm	3.90 ± 0.05	59.712 ± 13.79*^a^*	62.912 ± 9.59*^a^*	61.213 ± 13.61*^a^*	3.56 ± 0.05*^b^*	38.451 ± 6.58*^a,b^*	47.414 ± 4.95*^a,b^*	43.919 ± 5.29*^a,b^*
LVID;s, mm	2.83 ± 0.04	101.628 ± 21.67*^a^*	105.065 ± 15.16*^a^*	104.755 ± 19.54*^a^*	2.61 ± 0.08*^b^*	69.720 ± 8.39*^a,b^*	80.435 ± 8.576*^a,b^*	70.877 ± 9.57*^a,b^*
LVAW;d, mm	0.63 ± 0.01	0.41 ± 0.11*^a^*	0.56 ± 0.07	0.39 ± 0.08*^a^*	0.58 ± 0.01	0.41 ± 0.09*^a^*	0.54 ± 0.08	0.27 ± 0.03*^a^*
LVAW;s, mm	0.93 ± 0.01	0.56 ± 0.15*^a^*	0.68 ± 0.08*^a^*	0.48 ± 0.12*^a^*	0.83 ± 0.01	0.46 ± 0.11*^a^*	0.67 ± 0.09*^a^*	0.34 ± 0.05*^a^*
LVPW;d, mm	0.63 ± 0.01	0.58 ± 0.10	0.76 ± 0.06	0.63 ± 0.10	0.58 ± 0.008	0.72 ± 0.05	0.76 ± 0.02	0.73 ± 0.06
LVPW;s, mm	0.94 ± 0.01	0.67 ± 0.11	0.95 ± 0.08	0.77 ± 0.11	0.84 ± 0.01	0.87 ± 0.06	0.94 ± 0.03	0.87 ± 0.06
HR, bpm	488.48 ± 16.80	505.57 ± 16.39	527.32 ± 19.77	521 ± 13.30	508.86 ± 7.07	541.5 ± 15.53	529.40 ± 7.14	528.60 ± 18.38
SV, μL	35.87 ± 1.38	30.02 ± 4.30	31.87 ± 4.00	29.855 ± 1.68	27.99 ± 0.62*^b^*	24.30 ± 1.20*^b^*	29.48 ± 2.97*^b^*	23.43 ± 1.77*^b^*
CO, mL/min	17.48 ± 0.76	14.93 ± 1.91	16.71 ± 1.93	15.48 ± 0.77	14.25 ± 0.43	12.72 ± 0.57	15.61 ± 1.63	12.28 ± 0.62

Values are presented as mean ± SEM. Statistical analyses were performed using 2-way analysis of variance with sex and MI status (baseline vs post-MI) as factors, followed by Tukey post hoc test for multiple comparisons.

Abbreviations: bpm, beats per minute; CO, cardiac output; d, in diastole; EF, ejection fraction; FS, fractional shortening; HR, heart rate; LV, left ventricular; LVAW, left ventricular anterior wall; LVID, left ventricular internal diameter; LVPW, left ventricular posterior wall; LVVol, left ventricular volume; MI, myocardial infarction; s, in systole; SV, stroke volume.

Statistical significance is indicated as follows: *^a^P* less than .005 for post MI vs baseline within the same sex; *^b^P* less than .005 for the main effect of sex. LVID and LVVol values are expressed as the percentage change from each animal's individual baseline to account for sex-related differences in body size.

**Table 2.** Body and heart weight parameters in male and female mice at 12 weeks after myocardial infarction

Sex	Surgery	n	BW, g	HW, mg	HW/BW, mg/g
Male	Sham	4	26.418 ± 2.323	120.700 ± 17.370	4.638 ± 0.689
	12 wk MI	8	28.798 ± 0.807	167.143 ± 10.834*^a^*	5.798 ± 0.455*^a^*
Female	Sham	4	24.553 ± 2.399*^b^*	96.600 ± 6.972*^b^*	3.975 ± 0.207
	12 wk MI	8	23.691 ± 0.698*^b^*	119.363 ± 10.342*^a,b^*	5.030 ± 0.406*^a^*

Values are presented as mean ± SEM. Statistical analyses were performed using 2-way analysis of variance with sex and MI status (sham vs MI) as factors, followed by Tukey post hoc test for multiple comparisons.

Abbreviations: BW, body weight; HW, heart weight; HW/BW, heart-to-body-weight ratio; MI, myocardial infarction.

Statistical significance is denoted as follows: *^a^P* less than .05 for the main effect of MI and *^b^P* less than .05 vs for the main effect of sex.

**Figure 1.**
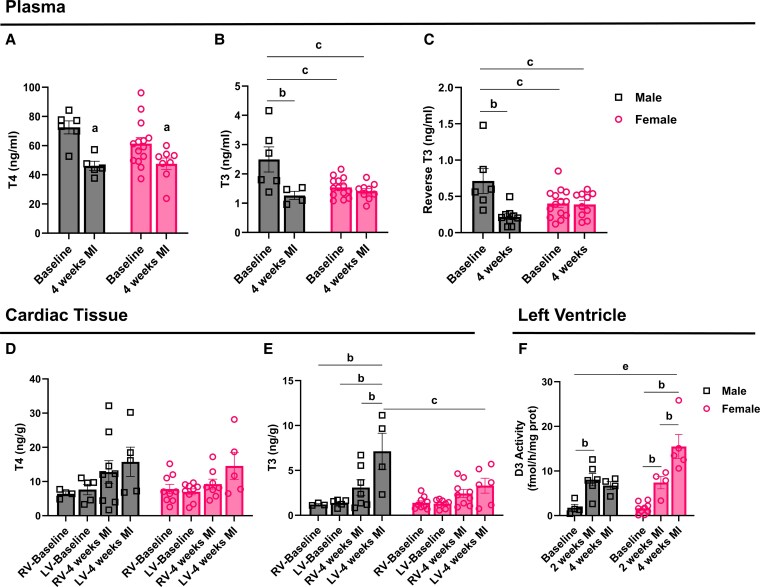
Plasma levels of A, thyroxine (T4); B, 3,5,3′-triiodothyronine (T3); and C, reverse T3 in males and females at baseline and 4 weeks post myocardial infarction (MI). D, Cardiac levels of T4 in the left ventricle (LV) and right ventricle (RV) of males and females at baseline and 4 weeks post MI. E, Cardiac levels of T3 in the LV and RV of males and females at baseline and 4 weeks post MI. F, D3 activity in the LV of males and females at baseline, 2 weeks, and 4 weeks post MI. Values are expressed as mean ± SEM, with individual data points shown. Statistical analyses were performed using 2-way analysis of variance with sex and MI status (baseline vs MI) as factors, followed by Tukey post hoc test for multiple comparisons. Statistical significance is indicated as follows: a, for the main effect of MI; b and c, for significant Tukey post hoc comparisons for MI and sex; and e, for significant Tukey post hoc comparisons between females at 4 weeks post MI and males across baseline, 2 weeks post-MI, and 4 weeks post-MI (*P* < .05).

We then assessed whether MI induced differences in plasma TH levels between male and female mice. While males and females had similar plasma levels of T4 at baseline (Fig. 1A), female mice exhibited significantly lower levels of T3 and rT3 compared to males (Fig. 1B and 1C). However, 4 weeks post MI, male mice showed significantly lower plasma T4, T3, and rT3 levels compared to baseline (see Fig. 1A-1C), whereas female mice exhibited only lower T4 levels (see Fig. 1A). Despite lower circulating levels of T4 and T3 following MI, male mice exhibited similar T4 levels and significantly higher T3 levels in the peri-infarct region of the LV compared to baseline (Fig. 1D and 1E). In addition, cardiac T3 levels in the LV were significantly higher than in the unaffected right ventricle (RV) (see Fig. 1E). In contrast, MI did not affect LV T4 levels in female mice (see Fig. 1D). Additionally, post-MI T3 levels in the LV remained unchanged in females and were significantly lower compared to males (see Fig. 1E). Notably, while cardiac D3 activity increased approximately 4-fold both in males and females at 2 weeks post MI, only females exhibited a further rise by 4 weeks, with D3 activity more than doubling from 7.4 to 15.49 fmol/h/mg protein. This sustained upregulation was not observed in males (Fig. 1F).

**Figure 2.**
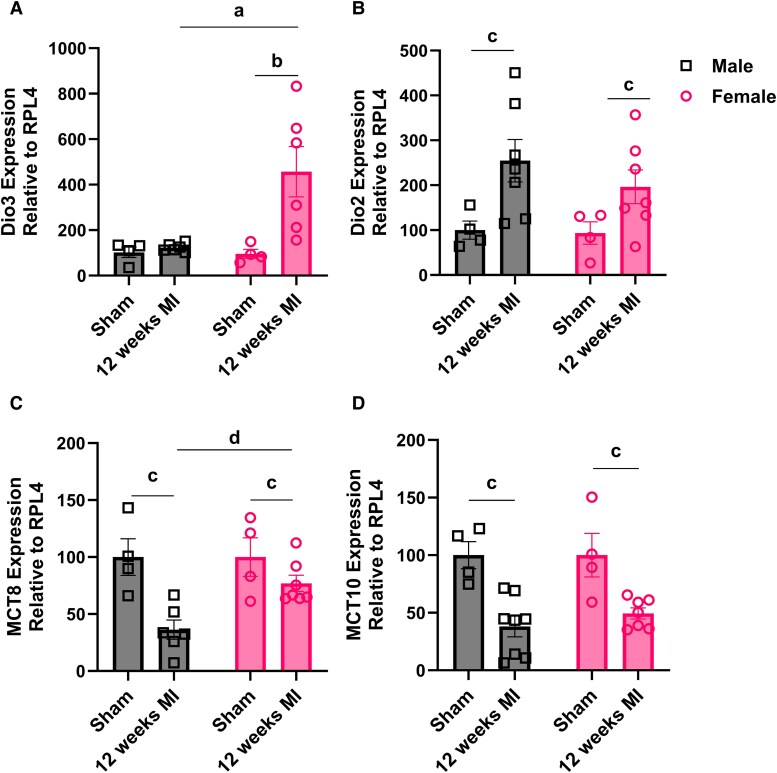
Relative gene expression of A, type 3 deiodinase (*Dio3*); B, type 2 deiodinase (*Dio2*); C, thyroid hormone transporter 8 (*Mct8*); and D, thyroid hormone transporter 10 (*Mct10*) in male and female mice subjected to sham surgery or 12 weeks post myocardial infarction (MI). Gene expression levels are normalized to the housekeeping gene *RPL4*. Values are expressed as mean ± SEM, with individual data points shown. Statistical analyses were performed using 2-way analysis of variance with sex and MI status (sham vs MI) as factors, followed by Tukey post hoc test for multiple comparisons. Statistical significance is indicated as follows: a and b, for significant Tukey post hoc comparisons for sex and MI; c, for the main effect of MI; d, for the main effect of sex (*P* < .05).

To determine whether TH regulation remains altered during late cardiac remodeling, we assessed mRNA levels of *Dio3*, *Dio2*, *Mct8*, and *Mct10* at 12 weeks post MI in male and female mice. *Dio3* mRNA expression was upregulated in the peri-infarct region of the LV of females, whereas in males, *Dio3* expression did not differ significantly from sham controls (Fig. 2A). In addition, we observed an upregulation of *Dio2*, the enzyme responsible for local T3 activation, both in males and females (Fig. 2B), along with a downregulation of the TH transporters *Mct8* and *Mct10* (Fig. 2C and 2D). Notably, the downregulation of *Mct8* was less pronounced in females, resulting in significantly higher expression levels compared to males (see Fig. 2C). Collectively, these findings point to a unique, female-specific mechanism of TH regulation following cardiac injury, in which sustained D3 upregulation may limit local T3 availability and alter the myocardial response to ischemic stress.

### Cardiomyocyte Type 3 Deiodinase Is Essential for Maintaining Local Thyroid Hormone Balance in Female Hearts Post Myocardial Infarction

**Figure 3.**
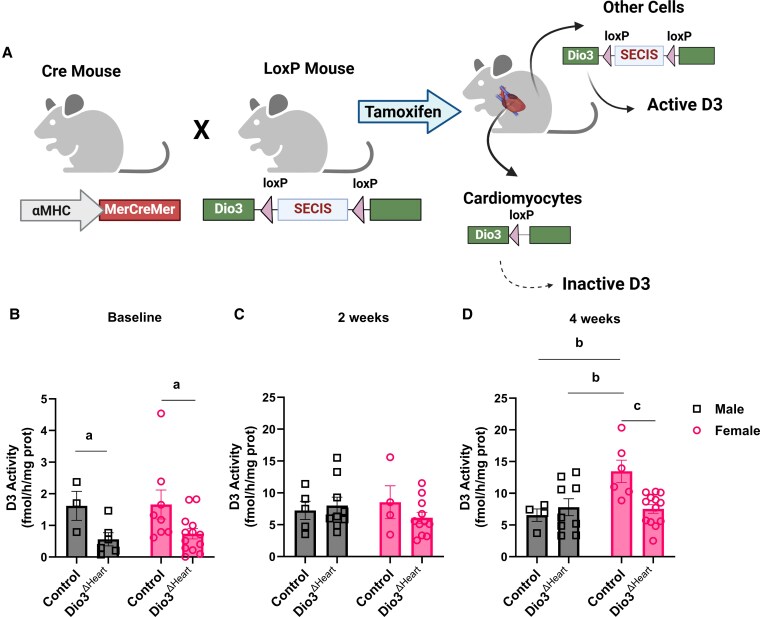
A, Schematic representation of the animal model. D3 activity in the left ventricle of control and *Dio3*^ΔHeart^ mice at B, baseline; C, 2 weeks; and D, 4 weeks post myocardial infarction (MI). Values are expressed as mean ± SEM, with individual data points shown. Statistical analyses were performed using 2-way ANOVA with genotype and sex as factors, followed by Tukey post hoc test for multiple comparisons. Statistical significance is indicated as follows: a, for the main effect of genotyping; b and c, for significant Tukey post hoc comparisons for sex, genotyping, and interactions (*P* < .05).

As we found that D3 activity in the peri-infarct region of the LV was approximately 2-fold higher in female mice compared to males at 4 weeks post MI (Fig. 1F), we next investigated whether this elevated D3 activity contributes to the sex-specific differences in systemic and cardiac TH levels following MI. To test this, we used mice with a cardiomyocyte-specific deletion of the SECIS in the *Dio3* gene, a noncoding sequence in the *Dio3* mRNA that directs the incorporation of selenocysteine into the active site of the protein and is critical for enzymatic function (genetic design is illustrated in Fig. 3A). As shown in Fig. 3B, this targeted deletion significantly reduced baseline D3 activity in both sexes, confirming successful specific disruption of D3 activity in cardiomyocytes.

At 2 weeks post MI, LV D3 activity was similarly higher across all groups, with no statistically significant differences between control and *Dio3*^ΔHeart^ mice of either sex (Fig. 3C). By 4 weeks post MI, control females showed a statistically significantly higher level of D3 activity (13.45 ± 1.76 pmol/min/mg; n = 6) compared to all other groups, including control males (6.54 ± 0.97 pmol/min/mg; n = 4), *Dio3*^ΔHeart^ males (7.80 ± 1.33 pmol/min/mg; n = 9) and females (7.52 ± 0.68 pmol/min/mg; n = 13). These groups did not appear to show further increases relative to their 2-week levels (control males: 7.24 ± 1.43 pmol/min/mg; *n* = 5; *Dio3*^ΔHeart^ males: 8.0 ± 1.32 pmol/min/mg; n = 9; control females: 8.5 ± 2.57 pmol/min/mg; n = 4; *Dio3*^ΔHeart^ females: 6.08 ± 0.86 pmol/min/mg; n = 11) (Fig. 3D). Given that our assay measures total tissue-level D3 activity and our genetic manipulation targets specifically cardiomyocytes, these findings suggest a dynamic shift in the cellular sources of D3 after MI. The robust increase in D3 activity observed in control females at 4 weeks post MI appears to be primarily driven by cardiomyocytes, as this elevation is blunted in *Dio3*^ΔHeart^ females (see Fig. 3D). In contrast, the less pronounced and genotype-independent D3 activity observed in both sexes at 2 weeks post MI and in males at 4 weeks post MI likely reflects contributions from noncardiomyocyte populations—such as endothelial cells (>60% of nonmyocytes), fibroblasts (∼20%), and hematopoietic-derived cells (5-10%)—which also express *Dio3* and respond to stress in a cell-type–specific manner (12-14, 27-29).

To determine whether loss of cardiomyocyte D3 activity affected overall cardiac mass, we compared BW, HW, and HW/BW ratios in male and female control and *Dio3*^ΔHeart^ mice at baseline, 2 weeks, and 4 weeks post MI (Table 3). While BW was lower in females than in males across all time points, MI induced a statistically significant increase in the HW/BW ratio in both sexes, regardless of genotype. No differences in HW or HW/BW were observed between control and *Dio3*^ΔHeart^ mice within the same sex, indicating that cardiomyocyte D3 deficiency did not influence gross cardiac hypertrophy.

**Table 3.** Body and heart weight parameters in male and female control and *Dio3*^ΔHeart^ mice at baseline and at 2 and 4 weeks after myocardial infarction

Sex	Surgery	Genotype	n	BW, g	HW, mg	HW/BW, mg/g
Male	Baseline	Control	7	30.176 ± 1.143	155.000 ± 6.729	5.169 ± 0.240
		*Dio3* ^ΔHeart^	7	25.234 ± 0.740	129.557 ± 3.562	5.146 ± 0.149
	2 wk MI	Control	12	27.582 ± 0.363	171.025 ± 8.697*^a^*	6.188 ± 0.293*^a^*
		*Dio3* ^ΔHeart^	10	26.598 ± 0.488	167.180 ± 5.574*^a^*	6.292 ± 0.208*^a^*
	4 wk MI	Control	14	27.136 ± 0.849	173.600 ± 6.642*^a^*	6.409 ± 0.198*^a^*
		*Dio3* ^ΔHeart^	14	27.724 ± 0.725	170.714 ± 6.764*^a^*	6.161 ± 0.192*^a^*
Female	Baseline	Control	17	23.022 ± 0.346*^b^*	118.506 ± 3.781*^b^*	5.142 ± 0.126
		*Dio3* ^ΔHeart^	15	22.299 ± 0.498*^b^*	110.613 ± 2.546*^b^*	4.973 ± 0.099
	2 wk MI	Control	10	21.564 ± 0.570*^b^*	147.470 ± 3.432*^a,b^*	6.869 ± 0.206*^a^*
		*Dio3* ^ΔHeart^	14	21.314 ± 0.467*^b^*	144.914 ± 6.577*^a,b^*	6.796 ± 0.259*^a^*
	4 wk MI	Control	19	22.079 ± 0.347*^b^*	151.447 ± 6.718*^a,b^*	6.858 ± 0.295*^a^*
		*Dio3* ^ΔHeart^	17	21.724 ± 0.334*^b^*	152.106 ± 7.005*^a,b^*	7.050 ± 0.346*^a^*

Values are presented as mean ± SEM. Statistical analyses were performed using 2-way analysis of variance with sex and MI status (baseline vs MI) as factors, followed by Tukey post hoc test for multiple comparisons.

Abbreviations: BW, body weight; HW, heart weight; HW/BW, heart-to-body-weight ratio; MI, myocardial infarction.

Statistical significance is denoted as follows: ^*a*^*P* less than .05 for the main effect of MI and ^*b*^*P* less than .05 for the main effect of sex.

**Figure 4.**
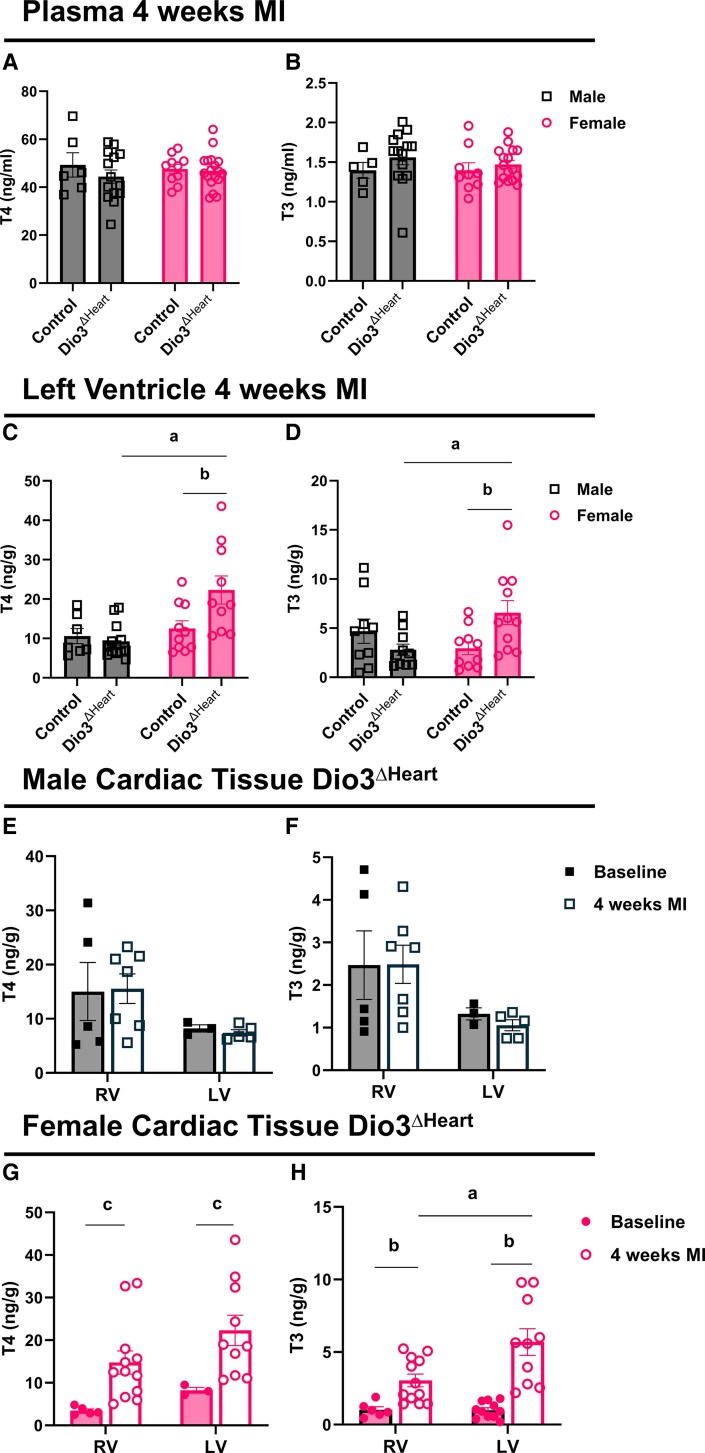
Plasma levels of A, thyroxine (T4) and B, 3,5,3′-triiodothyronine (T3) in male and female control and *Dio3*^ΔHEART^ mice at 4 weeks post myocardial infarction (MI). Left ventricle (LV) levels of C, T4 and D, T3 in male and female control and *Dio3*^ΔHEART^ mice at 4 weeks post MI. Right ventricle (RV) and LV levels of E, T4 and F, T3 in male *Dio3*^ΔHEART^ mice at baseline and 4 weeks post MI. Similarly, RV and LV levels of G, T4 and H, T3 are shown in female *Dio3*^ΔHEART^ mice at baseline and 4 weeks post MI. Values are expressed as mean ± SEM, with individual data points shown. Statistical analyses were performed using 2-way analysis of variance with sex and genotype as factors for panels A to D, and with MI status (baseline vs MI) and tissue (LV vs RV) as factors for panels E to H, followed by Tukey post hoc test for multiple comparisons. Statistical significance is indicated as follows: a and b for significant Tukey post hoc comparisons for sex and genotyping; c for the main effect of MI (*P* < .05).

Despite these differences in local D3 activity, plasma T4 and T3 levels remained unchanged between *Dio3*^ΔHeart^ and control groups in both sexes 4 weeks post-MI (Fig. 4A and 4B). However, while no differences were observed in LV T4 and T3 levels between *Dio3*^ΔHeart^ males and their control counterparts post MI, *Dio3*^ΔHeart^ females exhibited significantly higher LV T4 and T3 levels compared both to control females and *Dio3*^ΔHeart^ males (Fig. 4C and 4D). Notably, this increase in LV T3 levels in *Dio3*^ΔHeart^ females brought them to levels comparable to those observed in control males.

In *Dio3*^ΔHeart^ males, cardiac tissue T4 and T3 levels remained unchanged between baseline and 4 weeks post MI, with no statistically significant differences observed between the RV and LV (Fig. 4E and 4F). In contrast, *Dio3*^ΔHeart^ females exhibited significantly elevated tissue T4 levels both in the RV and in the peri-infarct region of the LV 4 weeks post MI (Fig. 4G). Notably, while tissue T3 levels in the RV showed an upward trend (*P* = .1118), this increase was not statistically significant. In contrast, T3 levels were significantly elevated in the peri-infarct region of the LV (Fig. 4H).

These findings indicate that cardiomyocyte-derived D3 plays a critical role in limiting local T3 accumulation in the female heart after MI.

### Reduced Cardiomyocyte Type 3 Deiodinase Activity Following Myocardial Infarction Impairs Cardiac Recovery in Female Mice

**Figure 5.**
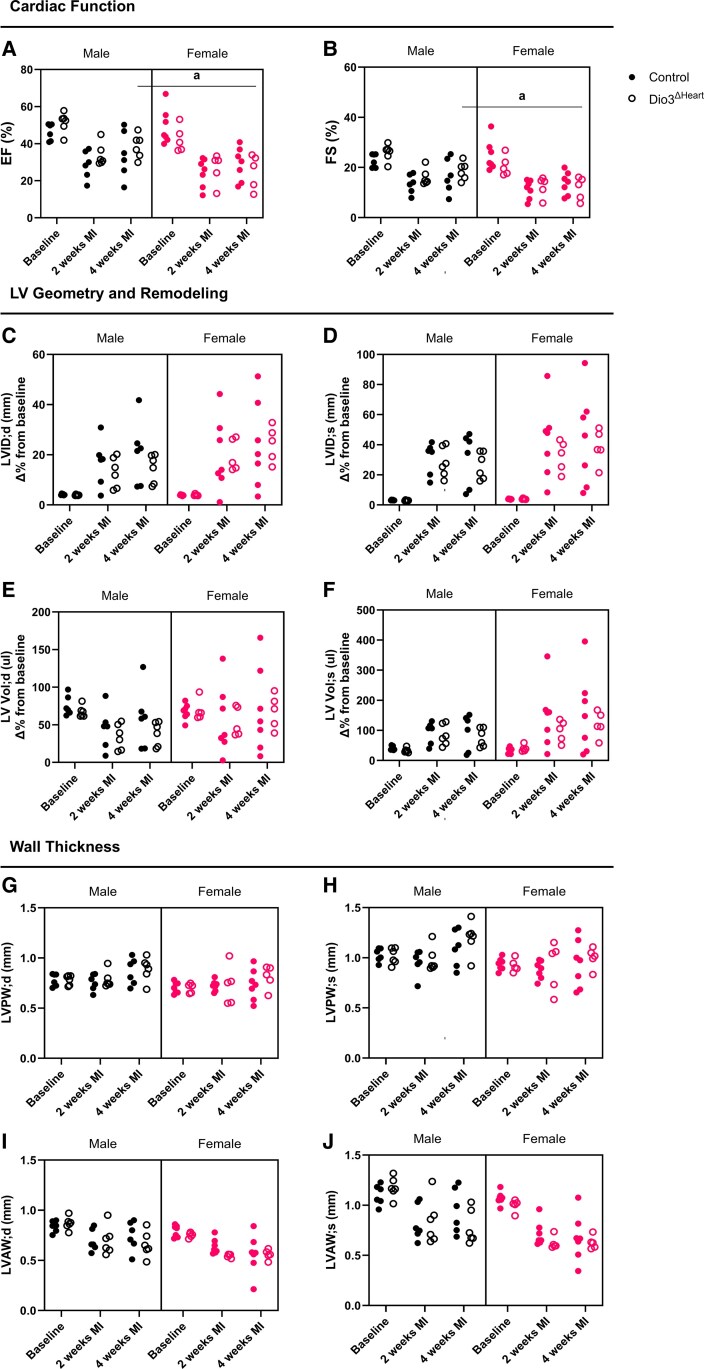
Assessment of cardiac function, left ventricular (LV) geometry, remodeling, and wall thickness by echocardiography. Echocardiographic parameters were evaluated in control and *Dio3*^ΔHeart^ male and female mice at baseline, 2 weeks, and 4 weeks post myocardial infarction (MI). Panels represent indices of i, cardiac function—A, ejection fraction (EF); and B, fractional shortening (FS); (ii) LV geometry and remodeling—C, LV internal diameter in diastole (LVID;d); D, LVID in systole (LVID;s); E, LV volume in diastole (LV Vol;d); F, LV in systole (LV Vol;s); and (iii) LV wall thickness—G, LV posterior wall thickness in diastole (LVPW;d); H, LVPW in systole (LVPW;s); I, LV anterior wall thickness in diastole (LVAW;d); and J, LVAW in systole (LVAW;s). For C to F, LVID and LVVol measurements, values are expressed as the percentage change from each animal's individual baseline to normalize for sex-related differences in body size. Echocardiographic images were acquired using the Vevo 2100 ultrasound system (VisualSonics). Data are presented as mean ± SEM, with individual data points shown. Statistical analyses were performed using a 3-way analysis of variance with sex, genotype, and MI status (baseline, 2 weeks, and 4 weeks post MI) as factors. Multiple comparisons were adjusted using the 2-stage linear step-up procedure of Benjamini, Krieger, and Yekutieli to control the false discovery rate. Statistical significance was set at *q* less than .05. Significance is indicated as follows: a, for the main effect of sex. MI produced significant effects both in males and females at 2 weeks and 4 weeks post MI for EF, FS, LVID, and LVAW; however, these symbols were omitted from the figure to avoid overcrowding the graphical presentation.

Given the elevated local T3 levels observed in *Dio3*^ΔHeart^ females post MI, we next evaluated whether reduced cardiomyocyte D3 activity affects cardiac function. Echocardiographic analyses were performed in *Dio3*^ΔHeart^ and control mice of both sexes at baseline, 2 weeks, and 4 weeks post MI. While no statistically significant genotype-dependent differences in EF or FS were observed over time within each sex (Fig. 5A and 5B), a sex-specific difference became evident at 4 weeks post MI: *Dio3*^ΔHeart^ females exhibited significantly lower EF and FS than *Dio3*^ΔHeart^ males (see Fig. 5A and 5B). These findings suggest that the absence of cardiomyocyte D3 activity hinders systolic recovery in females, although overall cardiac function was not significantly affected. Analysis of ventricular dimensions, expressed as the percentage change from each animal's individual baseline, revealed no statistically significant differences in LVID or LVVol among groups (Fig. 5C-5F). Similarly, no significant differences in anterior or posterior wall thickness were observed across groups (Fig. 5G-5J).

### Reduced Type 3 Deiodinase Activity Following Myocardial Infarction Lowers Lipid-Linked Mitochondrial Respiration in Female Mice

**Figure 6.**
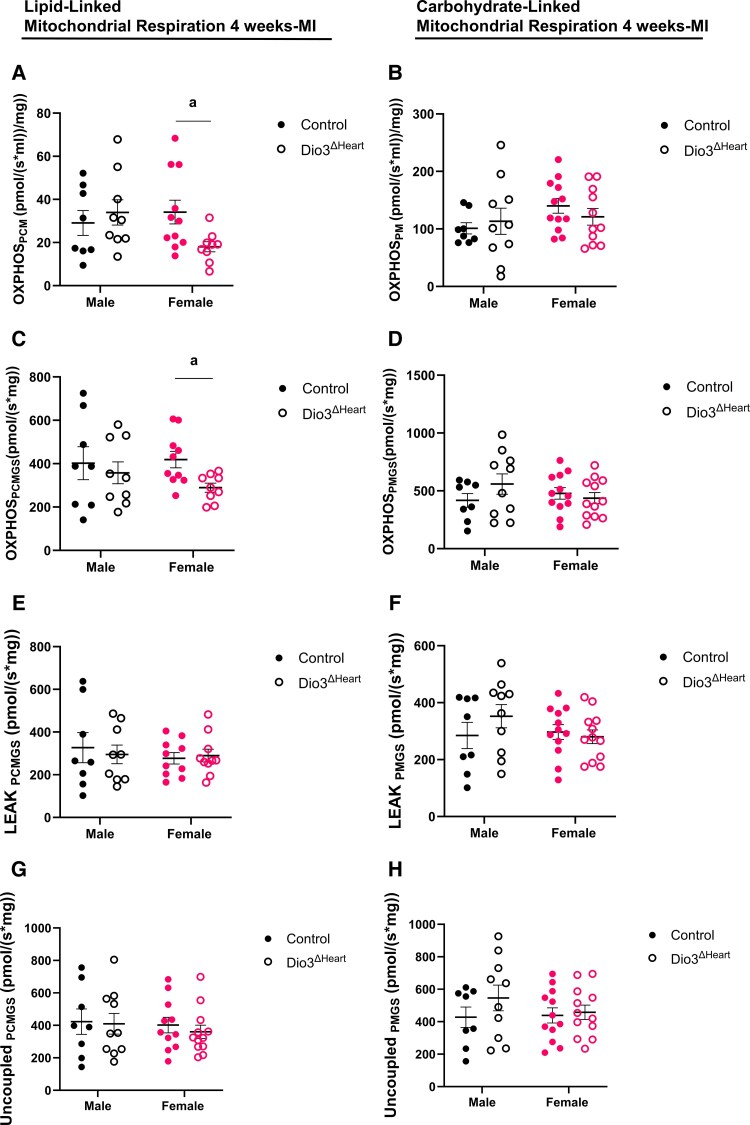
Mitochondrial respiration in control and *Dio3*^ΔHEART^ mice 4 weeks after myocardial infarction (MI). Oxygen consumption was assessed in permeabilized fibers from the peri-infarct region of the left ventricle (LV) using high-resolution respirometry (Oroboros O2k). Respiration was evaluated during A to D, oxidative phosphorylation (OXPHOS); E and F, LEAK state, and G and H, uncoupled state using A, C, E, and G, lipid-linked or B, D, F, and H, carbohydrate-linked substrates. Substrate combinations were PCM (P: palmitoylcarnitine; M: malate) and PCMGS (P: palmitoylcarnitine; M: malate; G: glutamate; S: succinate) for A, C, E, and G, lipid-linked panels, and B, D, F, and H, PM (P: pyruvate; M: malate) and PMGS (P: pyruvate; M: malate; G: glutamate; S: succinate) for carbohydrate-linked panels. Oxygen flux was normalized to wet tissue mass (pmol O₂·s⁻¹·mg⁻¹). Mitochondrial membrane integrity was verified by cytochrome *c* addition (<5% increase threshold). Data are presented as mean ± SEM, with individual data points shown. Statistical analyses were performed using multiple unpaired *t* tests, corrected for multiple comparisons with the Holm-Šidák method. Statistical significance is indicated by *a* (*P* < .05).

Given that functional capacity is tightly coupled to mitochondrial respiration, which supplies the adenosine triphosphate required for sustained contractile performance (30-33), we next assessed the effect of reduced cardiomyocyte D3 activity on mitochondrial respiration 4 weeks post MI. There were no statistically significant main effects or interactions between groups as analyzed by 2-way ANOVA. However, when comparing genotypes within each sex, oxidative phosphorylation (OXPHOS) capacity in response to lipid-linked substrates (OXPHOS__PCM_), and to combined lipid-linked Complex I and II substrates (OXPHOS__PCMGS_) was significantly lower in the peri-infarct region of the LV of female *Dio3*^ΔHeart^ mice compared to controls. No differences were observed between male *Dio3*^ΔHeart^ and control mice. Additionally, OXPHOS capacity in response to carbohydrate-linked substrates (OXPHOS__PM_) and to combined carbohydrate-linked complex I and II substrates (OXPHOS__PMGS_) did not differ by genotype or sex (Fig. 6A-6D).

LEAK respiration in response to lipid- or carbohydrate-linked substrates showed no statistically significant differences between control and *Dio3*^ΔHeart^ mice in either sex (Fig. 6E and 6F). Similarly, uncoupled respiration in response to lipid- or carbohydrate-linked substrates was comparable between groups (Fig. 6G and 6H). Lastly, no significant differences were observed in the LEAK/OXPHOS (L/P) or OXPHOS/Max Uncoupled (P/E) ratios under lipid-supported conditions. In contrast, the L/P ratio during carbohydrate-supported respiration was significantly elevated in the *Dio3*^ΔHeart^ male group compared to all other groups, suggesting impaired mitochondrial coupling efficiency in the male *Dio3*^ΔHeart^ group (Table 4).

**Table 4.** Ratios of mitochondrial respiration in permeabilized left ventricular fibers from male and female control and *Dio3*^ΔHeart^ mice 4 weeks after myocardial infarction

	Male		Female	
Parameters	Control	*Dio3* ^ΔHeart^	Control	*Dio3* ^ΔHeart^
Lipid PCMGS L/P	0.797 ± 0.041	0.820 ± 0.014	0.780 ± 0.033	0.764 ± 0.026
Lipid PCMGS P/E	0.952 ± 0.032	0.997 ± 0.020	0.989 ± 0.021	0.990 ± 0.026
Carbohydrate PMGS L/P	0.670 ± 0.025	1.00 ± 0.035*^a,b^*	0.641 ± 0.028	0.665 ± 0.027
Carbohydrate PMGS P/E	0.982 ± 0.014	1.00 ± 0.035	1.00 ± 0.035	0.958 ± 0.052

The table reports the ratios of LEAK to oxidative phosphorylation (OXPHOS) (L/P) and OXPHOS to maximal uncoupled respiration (P/E) under lipid-linked (PCMGS: palmitoylcarnitine, malate, glutamate, succinate) and carbohydrate-linked (PMGS: pyruvate, malate, glutamate, succinate) substrate conditions in male and female mice. Data are expressed as mean ± SEM in units of pmol O₂·s⁻¹·mg⁻¹. Statistical analyses were performed using 2-way analysis of variance with sex and genotype as factors, followed by Tukey post hoc test for multiple comparisons.

Statistical significance is denoted as follows: *^a^P* less than .05 for the main effect of genotype and *^b^P* less than .05 for the main effect of sex.

Together, these findings reveal a previously unrecognized role for cardiomyocyte D3 in supporting mitochondrial lipid oxidation during cardiac recovery in females. Loss of D3 activity impairs lipid-supported respiration post MI, potentially limiting energy availability and contributing to the sex-specific reduction in cardiac functional capacity observed in females compared to males.

### Cardiomyocyte Type 3 Deiodinase Deficiency Impairs Cardiac Function Without Affecting Infarct Size, Hypertrophy, or Survival in Female Mice

**Figure 7.**
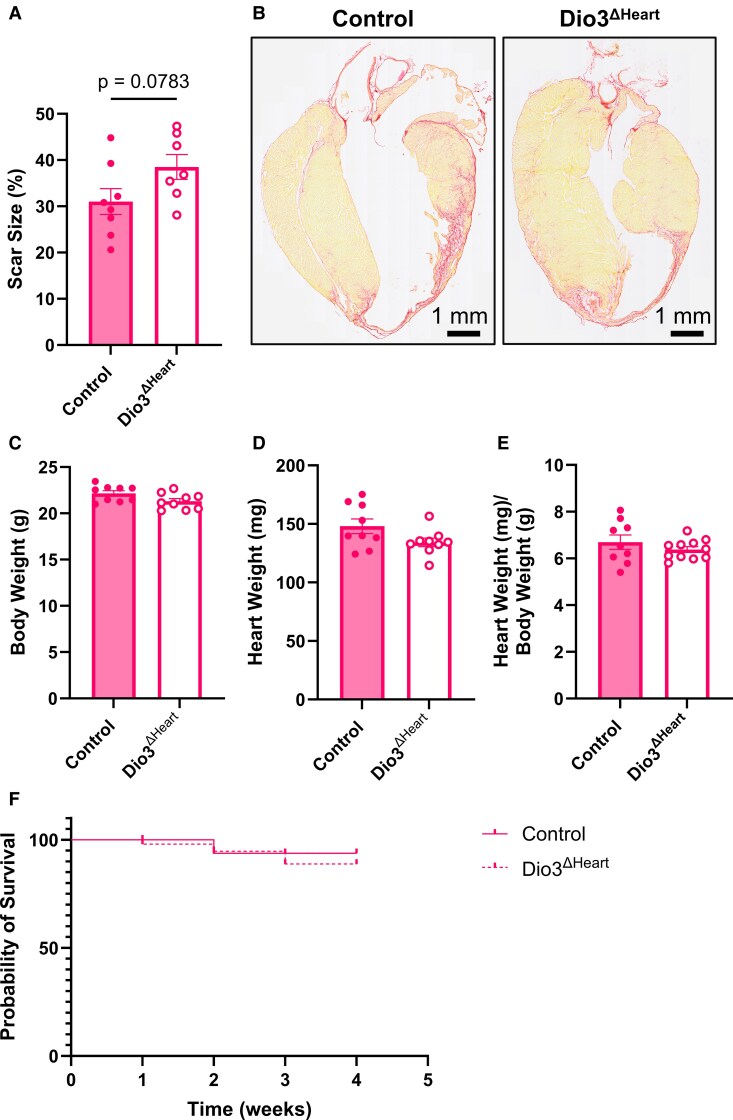
Assessment of scar expansion, gravimetric parameters, and survival rates post myocardial infarction (MI). Infarct scar circumference was evaluated in control and *Dio3*^ΔHeart^ female mice 4 weeks post MI. MI scar was visualized using Picrosirius red staining of histological sections and quantified as A, a percentage of scar circumference over the total left ventricular circumference. Representative Picrosirius red-stained sections from control and *Dio3*^ΔHeart^ female mice post MI are shown in B. Scale bars = 1 mm. At humane killing, 4 weeks post MI, C, body weight (BW); D, total heart weight (HW); and E, HW/BW ratios were measured in control and *Dio3*^ΔHeart^ female mice. Kaplan-Meier survival analysis was performed to assess post MI survival in F, wild-type (WT) and *Dio3*^ΔHeart^ mice. The solid line represents control mice, while the dashed line represents *Dio3*^ΔHeart^ mice. Probability of survival is plotted over time (weeks), with vertical tick marks indicating censored subjects. *P* = .6768; WT, n = 10; *Dio3*^ΔHeart^, n = 10. For A and C to E, data are shown as mean ± SEM, with individual data points plotted. Statistical analyses were performed using unpaired *t* tests (*P* < .05).

Given the reduced mitochondrial capacity observed in *Dio3*^ΔHeart^ females following MI, we next investigated whether the absence of cardiomyocyte D3 activity also affected infarct scar remodeling. Scar size was assessed in Picrosirius red–stained sections from *Dio3*^ΔHeart^ and control female mice at 4 weeks post MI. While no statistically significant difference in relative scar size was detected, a trend toward increased scar expansion was observed in *Dio3*^ΔHeart^ mice (*P* = .0783) (Fig. 7A and 7B), suggesting a potential influence of D3 on myocardial repair. Mortality rates and cardiac hypertrophy, assessed by the HW/BW ratio, were also comparable between genotypes at 4 weeks post MI (Fig. 7C–7F). Collectively, these findings indicate that loss of cardiomyocyte D3 activity in females primarily impairs functional recovery after MI without significantly altering structural remodeling or survival. While the functional impairment was female-specific, histological remodeling was not evaluated in males in this cohort; therefore, potential structural sex differences cannot be inferred.

### *DIO3* Expression Is Selectively Upregulated in Human Female Ischemic Cardiomyopathy Hearts

**Figure 8.**
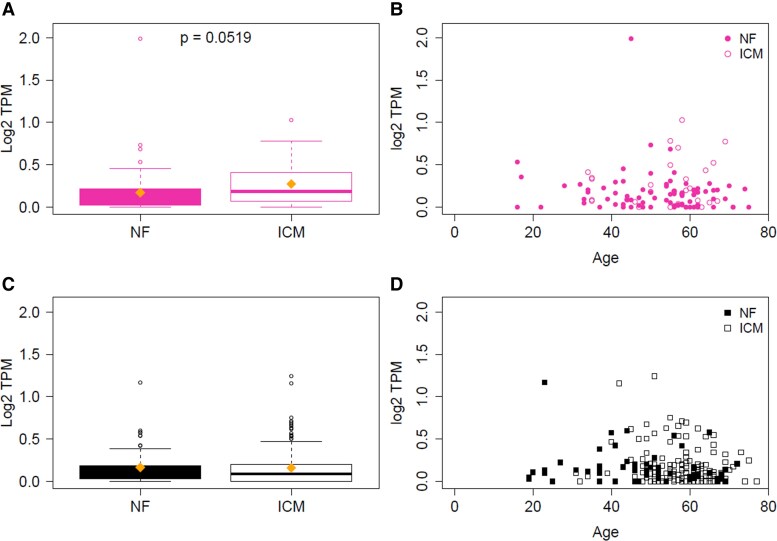
*DIO3* expression in human left ventricular tissue stratified by sex and disease status. The Y-axis shows log₂-transformed transcripts per million (TPM) values of *DIO3* (ENSG00000197406.7) in nonfailing (NF) and ischemic cardiomyopathy (ICM) human hearts. Data are presented separately for A and B, females and C and D, males. A and C, Left panels: Box plots display group medians, interquartile ranges, and means (indicated by diamonds). B and D, Right panels: Scatter plots show age vs log₂ TPM expression. In males, mean log₂ TPM was similar between NF (0.17) and ICM (0.16) hearts (fold change = −0.008; *P* = .24, Wilcoxon test). In females, mean log₂ TPM was higher in ICM (0.27) compared to NF (0.17) hearts (fold change = 0.10; *P* = .052, Wilcoxon test). Spearman correlation analyses showed no statistically significant association between age and *DIO3* expression in any group (female NF: ρ = −0.14; *P* = .22; female ICM: ρ = 0.02; *P* = .92; male NF: ρ = −0.21; *P* = .11; male ICM: ρ = −0.03; *P* = .72).

To assess the clinical relevance of our preclinical findings, we analyzed *DIO3* gene expression (ENSG00000197406.7) in LV tissue from male and female human donors with NF or ICM hearts across a broad age range. In females, *DIO3* expression was low and stable in NF hearts but significantly elevated in ICM hearts (Fig. 8A). However, no statistically significant correlation was observed between *DIO3* expression and age (Fig. 8B). In contrast, *DIO3* expression in male hearts did not differ between NF and ICM groups (Fig. 8C) and likewise showed no correlation with age (Fig. 8D). Together with our murine data, these results support a model in which post-injury *DIO3* induction serves as a sex-specific adaptive mechanism to regulate TH metabolism following ischemic damage.

## Discussion

Clinical and preclinical studies consistently report that reduced plasma T3 levels are associated with adverse outcomes following MI, including progression to HF (34-37). However, TH regulation after MI remains poorly understood (38-40), particularly in females, despite their higher prevalence of thyroid-related disorders (4, 41). Our study provides novel insights into sex-specific differences in cardiac TH metabolism after MI, demonstrating that although male mice experience a statistically significant drop in plasma T3, consistent with previous studies (36, 42), female mice maintain stable circulating T3 levels. Instead, females exhibit a robust, 2-fold increase in LV D3 activity compared to their male counterparts, suggesting a distinct sex-specific mechanism of TH regulation following MI.

The upregulation of D3 in female hearts appears to serve a sex-specific adaptive role that supports cardiac recovery following MI. In mice, females with cardiomyocyte-specific loss of D3 activity (*Dio3*^ΔHeart^) exhibited significantly lower systolic function than *Dio3*^ΔHeart^ males, despite no statistically significant differences in overall cardiac function, infarct size, cardiac hypertrophy, or survival compared to their respective controls. These findings suggest that D3 activity plays a functional role in preserving systolic performance in females after MI, independent of structural remodeling.

*Dio3* ^ΔHeart^ females exhibited significantly elevated T3 levels in the peri-infarct region of the LV post MI, which coincided with reduced oxidative phosphorylation capacity in response to fatty acid substrates, suggesting that D3 acts to limit T3-driven metabolic stress in the female heart. In contrast, *Dio3*^ΔHeart^ males showed no statistically significant changes in cardiac function, TH levels, or fatty acid– or carbohydrate-supported mitochondrial respiration, indicating a less prominent role for cardiomyocyte D3 in male cardiac recovery. Interestingly, the L/P ratio during carbohydrate-supported respiration was significantly elevated in *Dio3*^ΔHeart^ males compared to all other groups, suggesting impaired mitochondrial coupling efficiency in this context. Furthermore, while male WT mice typically undergo greater post-MI dilation than females, as indicated by higher percentage changes in LVID and LV volumes (25, 26), this pattern was blunted in *Dio3*^ΔHeart^ animals. D3-deficient males showed no significant differences in LVID and LV volumes compared to their controls or to *Dio3*^ΔHeart^ females. Together, these findings highlight a critical, sex-dependent role for cardiomyocyte D3 in modulating T3-driven metabolic changes after MI, potentially supporting recovery in females.

Our data suggest that D3 activity following MI evolves over time and shifts across cell types. Early after MI, the increase in D3 activity observed in both sexes is genotype-independent, suggesting that noncardiomyocyte populations, such as endothelial cells, fibroblasts, or immune cells, contribute to this response (12-14, 27-29). However, by 4 weeks post MI, a distinct, sex-specific pattern emerges, and only females exhibit a further increase in D3 activity, which is dependent on cardiomyocytes, as this increase is blunted in *Dio3*^ΔHeart^ females. This finding suggests that female cardiomyocytes are uniquely capable of upregulating D3 activity in response to prolonged stress, potentially as an adaptive mechanism to limit local T3 signaling and prevent metabolic overload. The absence of this response in *Dio3*^ΔHeart^ females underscores the essential role of cardiomyocyte D3 in female-specific myocardial adaptation during the chronic phase post MI.

While reduced plasma T3 levels after MI, observed in approximately 35% of patients (43, 44), are often attributed to increased extrathyroidal inactivation via D3 (34, 45, 46), our data suggest that this mechanism operates differently in females. Despite a robust upregulation of D3 activity in the LV of female mice, circulating T3 levels remain unchanged post MI, indicating that cardiomyocyte D3 upregulation does not affect systemic TH levels. Instead, this increase in cardiac D3 activity is associated with a sex-specific reduction in local T3 levels in the female heart, as females exhibit significantly lower LV T3 concentrations compared to males. This relationship is further supported by the finding that *Dio3*^ΔHeart^ females exhibit significantly higher LV T3 levels post MI, reaching levels comparable to those observed in males, confirming that D3 in cardiomyocytes specifically limits local T3 availability in the female heart.

Furthermore, while prior studies have demonstrated a positive correlation between circulating and tissue TH levels across multiple organs (47, 48), our findings show elevated LV T3 levels in male mice despite reduced plasma T3 following MI. This is consistent with previous reports of increased cardiac T3 concentrations in patients with low T3 syndrome post MI, and in animal models of dilated cardiomyopathy (48-51). In contrast to earlier studies in males suggesting that increased cardiac D3 activity reduces myocardial T3 levels (46, 51), we found that the modest D3 upregulation observed in male mice does not correspond with decreased LV T3. These discrepancies probably reflect differences in experimental models or time points of analysis after the injury. For example, our study used coronary artery ligation and measured cardiac T3 levels 4 weeks post MI, whereas previous studies used chronic adrenergic stimulation or assessed T3 levels at earlier stages post injury (46, 51).

Importantly, our findings suggest a more complex and sex-specific regulatory network governing cardiac TH homeostasis following MI. We observed statistically significant downregulation of *Mct8* in the LV, more pronounced in males than in females. As *Mct8* facilitates bidirectional transport of T4, T3, rT3, and 3,3′-T2 across the plasma membrane (52-54), its reduced expression may limit TH influx, particularly of T4, into cardiomyocytes. In parallel, *Dio2* expression was upregulated post MI, likely functioning as a compensatory mechanism to sustain intracellular T3 levels through local conversion of T4. Additionally, we observed downregulation of *Mct10*, which prefers T3 over T4 and may therefore reduce T3 efflux, promoting intracellular T3 retention (55). In females, this coordinated response is further regulated by *Dio3* upregulation, which fine-tunes intracellular T3 availability, balancing the need to prevent excessive TH signaling while supporting energy homeostasis and promoting cardiac recovery.

We speculate that the precise regulation of cardiac T3 levels via D3 activity is essential for heart recovery in females following MI. T3 enhances cardiac contractility and relaxation by regulating key proteins such as Serca2 and phospholamban (56, 57), thereby increasing HR and cardiac output. However, prolonged T3-driven upregulation of Serca2 increases myocardial energy demand, which can deplete adenosine triphosphate stores, impair calcium reuptake, and compromise cardiac efficiency (58). This mechanism is consistent with our findings in cardiomyocyte-specific D3-deficient female mice, which exhibited significantly elevated LV T3 levels and impaired systolic recovery 4 weeks post MI. These data suggest that insufficient T3 catabolism in the female heart leads to metabolic inefficiency and may increase vulnerability to HF.

We further propose that this T3-induced energetic imbalance in females disrupts myocardial energy homeostasis by impairing fatty acid oxidation, the heart's primary energy source under normal conditions (59-63). Maity et al (58) showed that prolonged T3 exposure in female rats suppresses both mRNA and protein levels of PPARα, a key transcription factor that promotes mitochondrial fatty acid oxidation. These findings suggest that prolonged T3 signaling impairs fatty acid metabolism in the female heart. Consistent with these findings, our data show that female mice with deficiency in cardiomyocyte D3 activity, which results in increased tissue T3 levels, exhibited significantly reduced lipid-linked mitochondrial respiration 4 weeks post MI compared to controls. Together, these results extend the findings of Maity et al (58), highlighting the importance of D3-mediated T3 inactivation in preserving mitochondrial fatty acid oxidation and metabolic homeostasis in the female myocardium.

Remarkably, our human data further support a sex-specific regulation of *DIO3* in response to cardiac injury. Transcriptomic analysis of LV tissue from NF and ICM hearts revealed a selective increase in *DIO3* expression in females with ICM. This upregulation was absent in males, whose *DIO3* levels remained stable regardless of disease status. These results are consistent with our murine findings, which demonstrate a sustained post-MI increase in cardiac D3 activity in females but not in males, highlighting a potential conserved mechanism of sex-specific TH inactivation. Importantly, because the human ICM samples were collected at variable time points during the HF phase following MI, the observed *DIO3* elevation in females may reflect a persistent or chronic adaptation to ischemic stress rather than an acute response.

Finally, it is important to consider that *DIO3* is an imprinted gene, predominantly expressed from the paternal allele (64, 65). Its expression can exhibit sex-specific regulation, often driven more by hormonal cues than by sex chromosome complement (66). In mice, estrogen stimulates *Dio3* expression in the uterus, supporting a hormone-dependent mechanism of regulation (67). However, in our human dataset, *DIO3* expression in females did not correlate with age, suggesting that increased expression in ICM is not affected by age-related changes in estrogen status. This divergence from the murine model may reflect tissue-specific and species-specific regulatory mechanisms or compensatory pathways that activate *DIO3* independently of estrogen. Notably, the small number of premenopausal female patients (aged <50 years) in our cohort may have limited our ability to detect estrogen-related differences earlier in life. Further studies are needed to define the hormonal and injury-responsive pathways that regulate D3 activity in the female heart.

Our findings reveal a previously unrecognized, female-specific mechanism of TH regulation in the injured heart. Cardiomyocyte D3 activity plays a critical role in shaping cardiac metabolic recovery by limiting T3-induced energetic stress and preserving lipid-supported mitochondrial respiration. These insights open new avenues for sex-specific therapeutic strategies that target TH metabolism to improve outcomes following cardiac injury.

### Limitations of the Work

Despite its strengths, this study has limitations that should be considered when interpreting the findings. Scar size and cardiac hypertrophy were quantified only in females; thus, conclusions about structural remodeling in males cannot be drawn. Nonetheless, our sex-specific interpretation is supported by functional and metabolic data and by female-selective *DIO3* upregulation in human ICM.

The cardiomyocyte-specific SECIS deletion model targets *Dio3* in myocytes; however, the heart also contains fibroblasts, endothelial, and immune cells that express *Dio3* and may influence total cardiac D3 activity after injury. Because our assay measures total LV D3 activity, contributions from nonmyocyte populations cannot be fully distinguished and may underlie temporal differences observed post MI.

Finally, we did not detect statistically significant genotype-specific differences in EF or FS within each sex. This may reflect the reduced power of the 3-way ANOVA, which was optimized to test sex × genotype × MI interactions, or compensation by residual *Dio3* expression in nonmyocyte cells. Such effects could attenuate detectable genotype-dependent changes in cardiac function. Nevertheless, our results strongly support that loss of cardiomyocyte D3 activity selectively impairs cardiac functional recovery in females, underscoring a sex-specific protective role for D3 in post-MI adaptation.

## Data Availability

All the murine data generated during and/or analyzed during the present study are available from the corresponding author on reasonable request. Human transcriptomic data analyzed in this study were obtained from the Trans-Omics for Precision Medicine (TOPMed) program. TOPMed omics datasets, including RNA sequencing, metabolomics, DNA methylation, and proteomics, are available through NIH-designated repositories. Specifically, data can be accessed via the database of Genotypes and Phenotypes (dbGaP; https://www.ncbi.nlm.nih.gov/gap) and BioData Catalyst (https://biodatacatalyst.nhlbi.nih.gov). Data dictionaries, variable summaries, and supporting documentation for the human RNA-seq dataset are publicly available via the dbGaP FTP site (study accession *phs002038.v1.p1*; https://ftp.ncbi.nlm.nih.gov/dbgap/studies/phs002038/phs002038.v1.p1). Access to full raw datasets requires dbGaP-approved projects and compliance with NIH data use policies. Due to restrictions related to human subject data, raw TOPMed datasets cannot be made publicly available, but can be requested through the appropriate controlled-access protocols. Additional information and deidentified data supporting the findings of this study are available from the corresponding author on reasonable request.

## Abbreviations

ANOVA: analysis of variance
bpm: beats per minute
BW: body weight
cDNA: complementary DNA
CO: cardiac output
d: in diastole
D3: type 3 deiodinase
*Dio3* ^ΔHeart^: cardiomyocyte-specific D3-deficient
EF: ejection fraction
FS: fractional shortening
HF: heart failure
HR: heart rate
HW: heart weight
ICM: ischemic cardiomyopathy
LC-MS/MS: liquid chromatography–tandem mass spectrometry
L/P: LEAK/OXPHOS
LV: left ventricular
LVAW: left ventricular anterior wall
LVID: left ventricular internal diameter
LVPW: left ventricular posterior wall
LVVol: left ventricular volume
MI: myocardial infarction
mRNA: messenger RNA
NF: nonfailing
OXPHOS: oxidative phosphorylation
PBS: phosphate-buffered saline
/E: OXPHOS/Max Uncoupled
RNA-seq: RNA sequencing
rT3: reverse T3 (3,3,5′-triiodothyronine)
RV: right ventricle
s: in systole
SECIS: selenocysteine insertion sequence
SV: stroke volume
T2: 3,3′-diiodothyronine
T3: 3,5,3′-triiodothyronine
T4: thyroxine
TH: thyroid hormone
WT: wild-type
